# *Mycobacterium tuberculosis* Inhibits RAB7 Recruitment to Selectively Modulate Autophagy Flux in Macrophages

**DOI:** 10.1038/srep16320

**Published:** 2015-11-06

**Authors:** Pallavi Chandra, Swapnil Ghanwat, Sumit Kumar Matta, Swati Seth Yadav, Mansi Mehta, Zaved Siddiqui, Amit Singh, Dhiraj Kumar

**Affiliations:** 1 Immunology Group, International Centre for Genetic Engineering and Biotechnology, Aruna Asaf Ali Marg, New Delhi, 110067, India; 2 Department of Microbiology and Cell Biology, Centre for Infectious Disease Research, Indian Institute of Sciences, Bangalore, 560012, India

## Abstract

Here we report a novel regulatory mechanism for autophagy-mediated degradation of *Mycobacterium tuberculosis (Mtb)* and specific strategy exploited by the virulent *Mtb* to evade it. We show while both avirulent (H37Ra) and virulent (H37Rv) mycobacteria could readily localize to autophagosomes, their maturation into autolysosomes (flux) was significantly inhibited by the latter strain. The inhibition of autophagy flux by the virulent strain was highly selective, as it did not perturb the basal autophagy flux in the macrophages. Selective inhibition of flux of *Mtb-*containing autophagosomes required virulence regulators PhoP and ESAT-6. We show that the maturation of *Mtb*-containing autophagosomes into autolysosomes required recruitment of the late endosome marker RAB7, forming the intermediate compartment amphisomes. Virulent *Mtb* selectively evaded their targeting to the amphisomes. Thus we report a crosstalk between autophagy and phagosome maturation pathway and highlight the adaptability of *Mtb,* manifested by selective regulation of autophagy flux.

Autophagy is a degradation process where cellular cargos are delivered to the lysosomes. Initially considered as a response activated under stress conditions, specifically nutrient stress, autophagy now is known to impact on diverse patho-physiological conditions like aging, neurodegenaration, inflammation and infection^1^,^2^. Recently the new paradigm of selective autophagy has emerged through which damaged cellular organelles or intracellular bacterial pathogens could be selectively targeted to autophagy for degradation. The ability to selectively recognize and target its cargo towards degradation has essentially led to the recognition of the autophagy pathway as an innate defense mechanism^3^. The autophagy of intracellular pathogen is termed as xenophagy while those of damaged organelle or other cellular cargo as macro-autophagy or simply autophagy^4^. Not surprisingly therefore, many intracellular pathogens including *Listeria*, *Legionella*, *Shigella*, *Salmonella*, *Coxiella*, *Group A Streptococcus* and *Mycobacteria* are known to specifically evade autophagy^5^.

Upon infection of macrophages with *Mycobacterium tuberculosis* (*Mtb*), the role of phagosome maturation pathway has been well elucidated^6^. Also known are the mechanisms leading to phagosome maturation arrest that help virulent *Mtb* escape from being targeted to lysosomes and killing^7^,^8^. For instance it has been convincingly shown that virulent *Mtb*-containing phagosomes do not acquire late endosome marker RAB7, leading to phagosomal maturation arrest^7^,^8^. Interestingly autophagy is yet another mechanism which can influence the targeting of *Mtb* to the lysosomes and subsequent killing. Despite several reports on the involvement of autophagy in regulation of *Mtb* killing^9^,^10^,^11^, how these two processes (autophagy and phagosome maturation) interact with each other and get perturbed by *Mtb* remains poorly understood.

Autophagy involves sequestration of cellular cargo in a double-membrane structure called autophagosomes. Autophagy induction is conventionally monitored as MAP1LC3B (LC3) puncta formation through microscopy. It emerges now, autophagy induction does not always lead to degradation of the cargo. Degradation typically involves fusion of autophagosomes with the lysosomes thereby forming autolysosomes and subsequent action of lysosomal hydrolases. However during the degradation process, LC3 on the inner membrane of autophagosomes too gets degraded due to the activity of lysosomal hydrolase^12^. Therefore when active autophagic degradation is taking place, LC3 would be constantly degraded leaving only the outer membrane associated LC3 intact and thereby making it difficult to accurately assess autophagy induction. The degradation of LC3 however can be blocked by vacuolar ATPase inhibitors like bafilomycin A1 or lysosomal protease inhibitors like E64-d and pepstatin A^12^. Strangely several studies in the past, which showed an important role for autophagy in case of *Mtb* infections, did not monitor autophagy flux^9^,^13^,^14^. In some studies however, in case of *Mtb* infections autophagy flux as a means of bacterial degradation were monitored^15^,^16^,^17^. One such report suggests strain dependent variations in autophagy induction which also correlated with the autophagic degradation^17^. Yet another study identified the immunity regulator molecule TBK1 as key regulator of autophagy maturation^15^.

In the present study we aimed to investigate the autophagy flux in THP-1 macrophages upon *Mtb* infection in detail using laboratory strains H37Ra (avirulent) and H37Rv (virulent). Interestingly both the virulent and avirulent strains localized to the autophagosomes. Later in the infection, the flux of *Mtb*-containing autophagosomes was specifically inhibited by the virulent *Mtb.* We then show that the inhibition of autophagy flux by H37Rv was the result of its ability to block recruitment of RAB7 on *Mtb*-containing autophagosomes. Therefore through this study we report a RAB7-dependent autophagy pathway of mycobacterial killing in macrophages.

## Results

### Virulent *Mycobacterium tuberculosis* selectively inhibits autophagy flux

**Figure 1:**
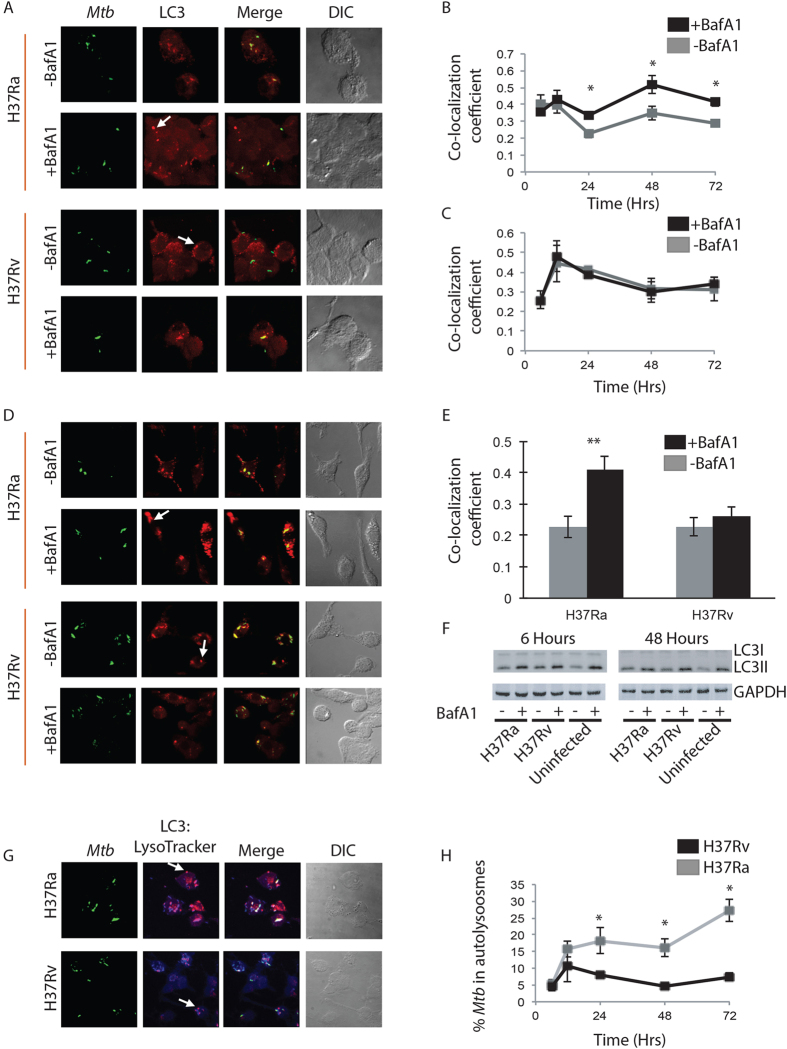
Virulent *Mycobacterium tuberculosis* selectively inhibits autophagy flux. PMA differentiated (20 ng/ml, 24 hours) THP-1 macrophages were infected with PKH67 (green) labelled H37Ra or H37Rv at MOI of 1:10 (methods). Autophagosomes were visualized by LC3 immuno-staining at 6, 12, 24, 48 and 72 hours post infection with and without BafA1 treatment (100 nM, 3 hr). (**A**) Representative images showing co-localization of both H37Ra and H37Rv (green) to autophagosomes (LC3, red) at 48 hours post infection. Panels (**B,C**) show Co-localization Coefficient M1 of *Mtb* and LC3 for H37Ra and H37Rv infection, respectively with and without BafA1 treatment (Mean ± Standard Error Measurements, *p < 0.05). Panel (**D**) shows representative images of *Mtb*-infected mouse BMDMs with LC3 (red) at 48 hours post infection with and without BafA1. Panel E shows the corresponding quantification of the dataset (mean ± SEM, **p < 0.01). Panel (**F**) shows LC3 immunoblots from the cell lysates of H37Ra or H37Rv infected macrophages as well as uninfected control macrophages at 6 and 48 hours post infection. Cells were treated with BafA1 (100 nM, 3 hr) to assess autophagy flux. Panel (**G**) shows representative images of *Mtb* in autolysosomes (LC3:LysoTracker; (blue:red)) at 48 hours post infection. The plot for the same at 6, 12, 24, 48 and 72 hours post infection is depicted in Panel (**F**) (Mean ± Standard Error Measurements, *p < 0.05).

The laboratory strains H37Rv and H37Ra of *Mtb*, consistent with previous reports, showed distinct survival ability within the macrophages (Fig. S1A)^18^,^19^,^20^. To check how autophagy gets regulated under the two conditions, we monitored targeting of H37Ra or H37Rv to LC3 positive autophagosomes by immuno-staining followed by confocal microscopy. THP-1 macrophages were infected with H37Ra or H37Rv at 1:10 MOI following the protocol described previously^10^. We monitored autophagosome targeting of the two strains at 6, 12, 24, 48 and 72 hours post-infection. Throughout the course of infection, both H37Ra and H37Rv efficiently co-localized with LC3 containing compartments (Fig. 1A). The antibody used to stain endogenous LC3 was highly specific and did not show any cross-reactivity with the *Mtb* (Fig. S1B and S1C). We calculated the M1 Coefficient with respect to *Mtb* (herein after referred to as co-localization coefficient, see methods) to measure co-localization of both the strains to autophagosomes. In parallel we also had groups where samples were treated with vacuolar ATPase inhibitor Bafilomycin A1 (BafA1, 100 nM) for three hours before the end of each of the time points. Treatment with BafA1 inhibits LC3 degradation upon maturation by blocking the acidification of autolysosomes. We observed a significant increase in the co-localization coefficient of H37Ra with LC3 in BafA1 treated samples relative to the untreated ones from 24 hours post infection (Fig. 1B). Importantly no such increase was observed in case of H37Rv across all time points (Fig. 1C).

We also tested these results in mouse bone marrow derived primary macrophages (BMDMs). Consistent with the results above, BafA1 treatment in BMDMs led to an increase in the co-localization coefficient of H37Ra and LC3, while there was no effect of BafA1 treatment in the case of H37Rv (Fig. 1D,E). Interestingly, Western blot analysis of LC3 in the whole cell lysates from H37Ra or H37Rv infected as well as uninfected THP-1 macrophages showed increased levels of lipidated LC3 (LC3II, Fig. 1F) upon BafA1 treatment, implying maturation of autophagosomes at certain basal state in these cells. We also noted both in THP-1 macrophages and in BMDMs, presence of autophagosomes that did not contain either H37Ra or H37Rv (arrows in Fig. 1A,D).

Differential flux of H37Ra and H37Rv containing autophagosomes was also confirmed by monitoring co-localization of *Mtb* with autolysosomes (Fig. 1G,H). Since BafA1 treatment diminishes LysoTracker staining of lysosomes, we used lysosomal protease inhibitors E64-d and pepstatin A to monitor H37Ra or H37Rv in LC3:LysoTracker double positive compartments representing autolysosomes. We observed a significantly higher percentage of H37Ra in autolysosomes, as compared to H37Rv, from 24 hour post infection (Fig. 1H). We also noted, in both the cases, presence of LC3:LysoTracker double positive compartments that did not contain any *Mtb* (Fig. 1G, arrows).

### *Mycobacterium tuberculosis* PhoP and ESAT-6 help H37Rv inhibit autophagy flux

**Figure 2:**
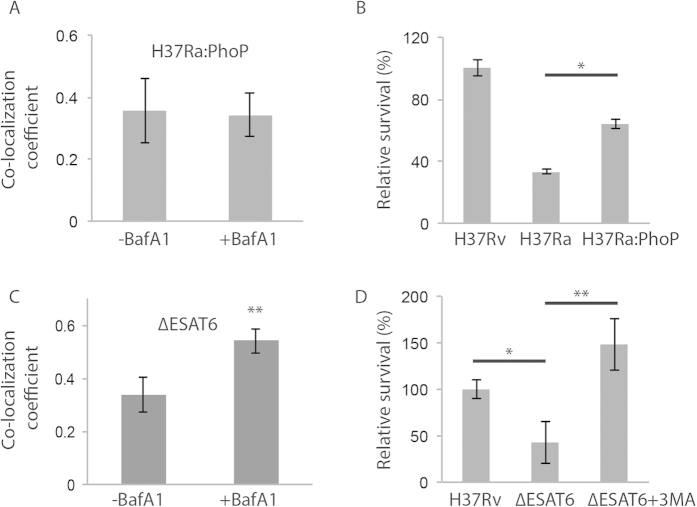
*Mycobacterium tuberculosis* PhoP and ESAT-6 help H37Rv inhibit autophagy flux. H37Ra was complemented with wild type *phoP* (called as H37Ra:PhoP strain). Panel A shows M1 co-localization coefficient of H37Ra:PhoP with LC3 in the presence or absence of BafA1 treatment, 48 hours post infection. Panel (**B**) shows relative survival percentage in THP-1 macrophages with respect to H37Rv, H37Ra or H37Ra:PhoP at 48 hours post-infection (mean ± SD, *p-value < 0.05). M1 co-localization coefficient of ∆ESAT-6 mutant of H37Rv with LC3 compartment in BafA1 treated and untreated condition is shown in 2C (mean ± SEM, **p < 0.001). In 2D, relative survival was estimated in H37Rv and ∆ESAT-6 infected THP-1 macrophages, 48 hours post-infection. The ∆ESAT-6 strain infected cells were treated with 3-MA (5 mM, 12 hours) to observe their rescue upon autophagy inhibition (mean ± SD, *p-value < 0.05, **p-value < 0.001).

The strain H37Ra differs from H37Rv mainly due to a point mutation in the master regulator gene PhoP^21^ and expression of wild-type (WT) PhoP (from H37Rv) in H37Ra is shown to revert the virulent phenotype^21^,^22^. We asked whether PhoP, a known *Mtb* virulence regulator, could help H37Rv modulate host xenophagy flux. We monitored co-localization of WT-PhoP complemented H37Ra strain (H37Ra:PhoP) with LC3 in THP-1 macrophages. PhoP complementation of H37Ra was confirmed by real-time analysis of several genes known to be regulated by PhoP (Fig. S2). The co-localization coefficient of H37Ra:PhoP with LC3 did not show any increase upon BafA1 treatment at 48 hours post infection (Fig. 2A). This response was similar to that of H37Rv at 48 hours post infection (Fig. 1A,C). Subsequently, H37Ra:PhoP had significantly higher intracellular survival with respect to H37Ra at 48 hours post infection (Fig. 2B). Partial rescue of the PhoP complemented strain further confirmed the association of autophagic flux with *Mtb* killing.

The *Mtb* PhoP is a global transcriptional factor involved in regulating the expression of RD1 region. H37Ra shows much diminished secretion of ESAT-6, the major antigen on RD1 locus and a well-known mycobacterial virulence effector^21^,^23^. To test if PhoP mediated block in the autophagy flux was effected by ESAT-6, we used ESAT-6 deleted mutant of H37Rv (∆ESAT-6). BafA1 treatment led to significant increase in the co-localization of ∆ESAT-6 strain with LC3 (Fig. 2C). To note here is that the block in autophagy flux observed in case of H37Rv was released with the deletion of ESAT-6. As expected, ∆ESAT-6 strain showed compromised survival in the THP-1 macrophages compared to the wild type H37Rv (Fig. 2D). Moreover treatment with 3-MA, an autophagy inhibitor, was able to rescue ∆ESAT-6 from killing (Fig. 2D).

### H37Rv inhibits amphisome formation

We next wanted to understand the cellular events in the host that gets perturbed by virulent *Mtb* in order to inhibit autophagy flux. Maturation of autophagosomes involves direct fusion with the lysosome to form auto-lysosome or they can first recruit RAB7 to form amphisomes which eventually fuse with the lysosomes^2^,^24^. It is well known that H37Rv inhibits RAB7 recruitment to escape lysosomal targeting and killing^8^. Therefore, we tested whether RAB7 recruitment on autophagosomes (amphisomes) could also represent a mechanism of H37Rv to evade lysosomal fusion. Therefore, we monitored co-localization of H37Ra and H37Rv to LC3-RAB7 double positive compartment (hereby defined as amphisomes) at 48 hours post-infection.

**Figure 3:**
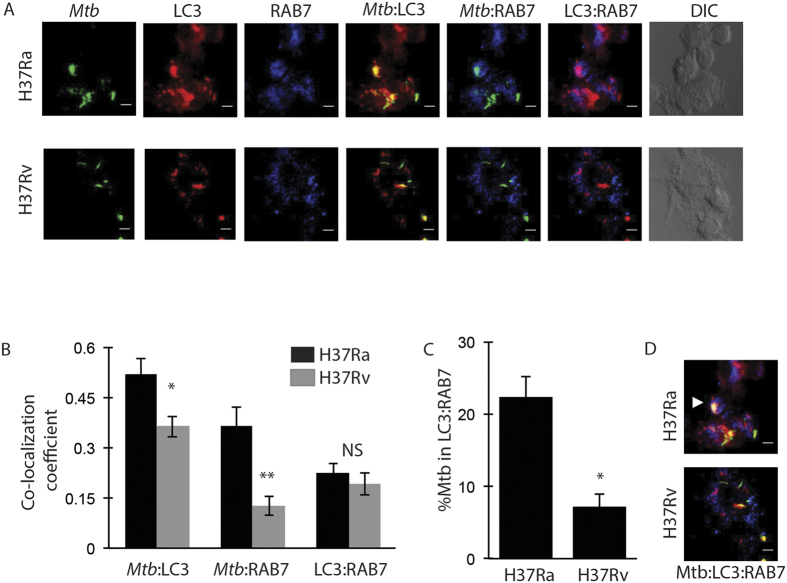
Virulent *Mtb* inhibits amphisome formation. Co-localization of H37Ra and H37Rv (green) to LC3 (red) and RAB7 (blue) were examined at 48 hours post-infection. Representative images are shown in panel (**A**). Panel B shows the plot of Co-localization Coefficient of *Mtb* to LC3 (Mtb:LC3), *Mtb* to RAB7 (Mtb:RAB7) and LC3 to RAB7 (LC3:RAB7) at 48 hours post infection (Mean ± SEM, *p < 0.05, **p < 0.001). Panel C shows percentage of *Mtb* in LC3-RAB7 amphisomes at 48 hours time point (Mean ± SEM, *p-value < 0.05). Panel (**D**) shows the representative images of H37Ra and H37Rv in amphisomes (white arrow). Scale bar represents 5 μm.

As expected we observed significantly higher co-localization of H37Ra with either LC3 or RAB7 as compared to H37Rv (Fig. 3A,B). Consistent with that, the percentage of H37Ra was significantly higher in amphisome (LC3:RAB7 double positive) as compared to H37Rv at 48 hours post infection (Fig. 3C). However, LC3-RAB7 co-localization, representing overall amphisome population in the cell, was similar for both the strains. These results clearly suggest that RAB7 recruitment to autophagosomes is significantly higher in H37Ra as compared to H37Rv. Therefore, we next tested whether the inhibition of autophagic flux by virulent *Mtb* is associated with RAB7 recruitment.

### Exclusion of RAB7 from *Mtb*-containing autophagosome allows H37Rv to escape lysosome targeting

**Figure 4:**
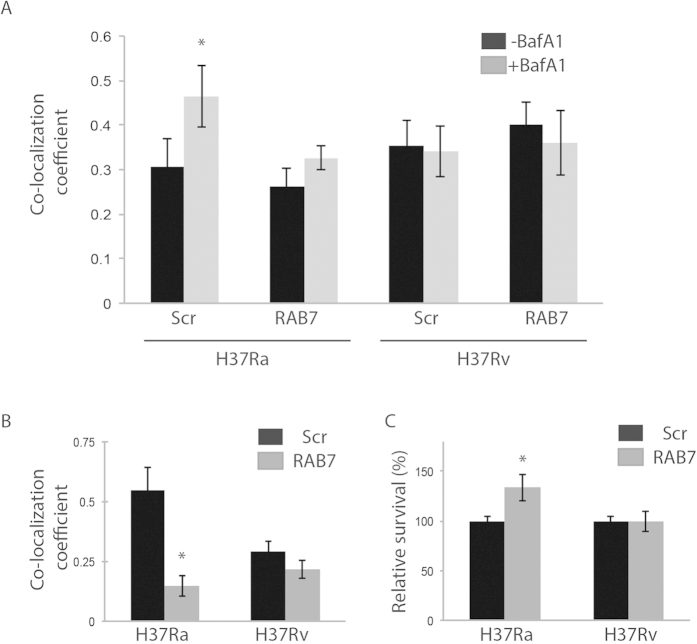
Exclusion of RAB7 from *Mtb*-containing autophagosome allows H37Rv to escape lysosome targeting. THP-1 macrophages infected with H37Ra or H37Rv were treated with specific siRNA against RAB7 (50 nM) till 48 hours post infection. Panel (**A**) shows co-localization coefficient M1 of H37Ra and H37Rv to autophagosomes in BafA1 treated and untreated conditions, with RAB7 siRNA treatment (RAB7) relative to control (SCR, scrambled) (mean ± SEM, *p < 0.05). Panel (**B**) shows co-localization coefficient M1 of H37Ra and H37Rv to LysoTracker (lysosomes) in the presence or absence of RAB7 siRNA treatment (mean ± SEM, *p < 0.05). Plot in panel C shows *Mtb* CFU with RAB7 siRNA treatment as compared to scrambled control.

In order to elucidate the role of RAB7 in modulating flux of *Mtb-*containing autophagosomes, specific siRNA was used to knockdown RAB7 in infected THP-1 macrophages. Specific knockdown of RAB7 was verified by Western blots (Fig S3A). As shown in Fig. 4A, RAB7 knockdown inhibited the flux of H37Ra-containing autophagosomes. There was no effect on flux of H37Rv-containing compartments (Fig. 4A). Intriguingly RAB7 siRNA also led to inhibition of general autophagy flux in THP-1 macrophages (Fig. S3B and S3C). As expected, co-localization of H37Ra to lysosomes was significantly inhibited in the RAB7 knockdown macrophages (Fig. 4B). Moreover RAB7 depletion also resulted in improved intracellular survival of H37Ra (Fig. 4C).

## Discussions

Autophagy as a defence mechanism in case of *Mtb* infection has been shown by several studies in the past^9^,^11^,^25^. Given the fundamental role of autophagy in cells as homeostatic mechanism under various stress conditions, how *Mtb* could specifically manipulate it, remains poorly understood. There are studies on strain specific regulation of autophagy, suggesting that the virulent strains of *Mtb* induce autophagy to a lesser extent in contrast to the non-pathogenic, attenuated strains^17^. Some other reports however suggest while the wild-type strains could induce autophagy, the attenuated ones could not^14^. Here we exclusively focused on the regulation of maturation of *Mtb*-containing autophagosomes and observed a distinct pattern of selectivity between the virulent and avirulent strains of *Mtb* in regulating autophagy flux.

We show here that both virulent and avirulent strains of *Mtb* can readily localize to the autophagosomes. Our experiments with BafA1 revealed a higher co-localization of H37Ra with autophagosomes relative to the untreated set post 24 hours of infection. Since BafA1 inhibits LC3 degradation in the autolysosomes, we believe the increase in co-localization was due to the enhanced signals from the accumulated, undegraded LC3 molecules in the autolysosomes. This result implies flux of H37Ra-containing autophagosomes that were maturing into autolysosomes. On the other hand, we did not observe any increase in the co-localization of H37Rv with autophagosomes upon BafA1 treatment throughout the course of infection, thereby suggesting a block in the flux of H37Rv-containing autophagosomes. This differential flux of *Mtb*-containing autophagosomes was corroborated by higher localization of H37Ra with the autolysosomes as compared to H37Rv. We found a crucial role of the virulence factors PhoP and ESAT-6 in effecting this maturation block of *Mtb*-containing autophagosomes. Similar role of ESAT-6 in the regulation of autophagy flux in infected dendritic cells has been shown earlier^16^.

Curiously, we observed several autophagosomes devoid of bacteria in the infected cells. Moreover, several of those autophagosomes were at maturation stage co-localizing with the acidified lysosomes. This observation indicated that the autophagosomes, which did not contain *Mtb,* could mature without being influenced by infection. The LC3 immunoblots provided additional evidence supporting the selective nature of maturation block brought by the virulent strain. This observation opens up an interesting possibility that non-*Mtb*-containing autophagosomes and *Mtb*-containing autophagosomes (xenophagosomes) could have distinct maturation dynamics within the same cell. A blanket inhibition of autophagy, under many circumstances, would lead to induction of apoptosis, which may be detrimental to *Mtb* survival^19^. By specifically blocking the maturation of compartments where H37Rv resides, it avoids being killed by lysosomal mechanism. At the same time by allowing cellular autophagy, it ensures extended survival of the host^26^,^27^. However, this interesting possibility deserves more detailed investigation.

**Figure 5:**
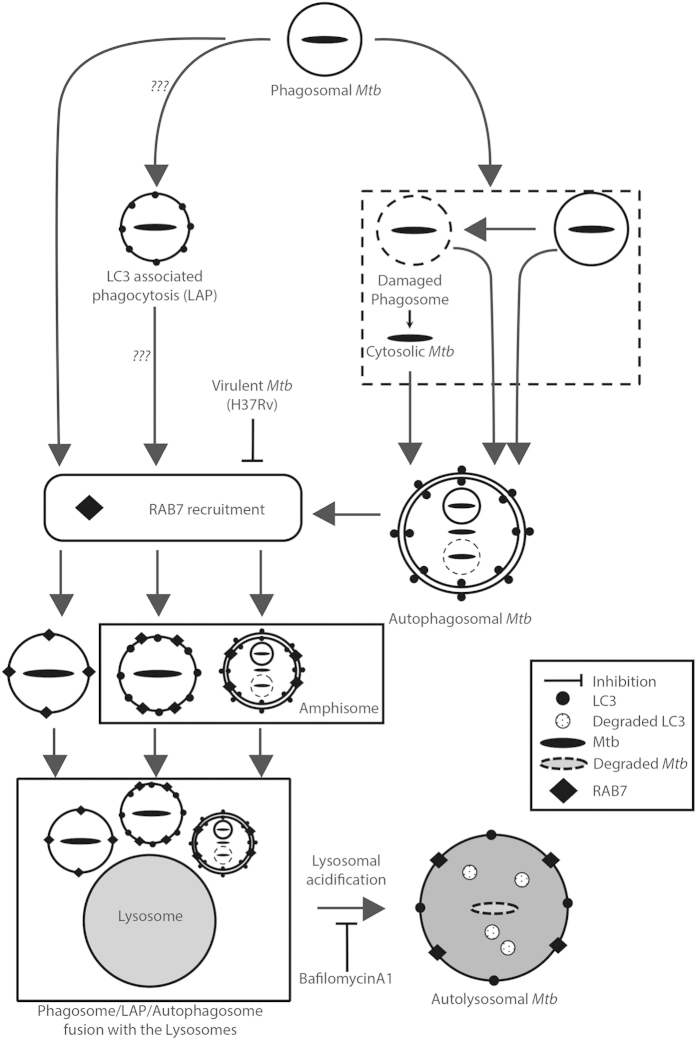
Revisiting *Mtb* phagosome maturation pathway. *Mtb* in the phagosomes could follow many pathways. Mtb, especially the virulent strains, is known to escape from the phagosome. Both phagosome bound and cytosolic *Mtb* may get trapped in the autophagosomes. Phagosome may also directly recruit LC3 to form a LAP vesicle. Further maturation of phagosomes, LAP or autophagosomes depends on recruitment of RAB7, which facilitates eventual fusion with the lysosome for degradation. Virulent strains of *Mtb* inhibit RAB7 recruitment on the phagosomes. This study shows, similar inhibition of RAB7 recruitment on autophagosomes and possibly on LAP by the virulent strain.

Our results on the role of RAB7 in autophagosome maturation provide novel insights into functioning of this small GTPase. Exclusion of RAB7 on phagosomes by virulent *Mtb* has been classically reported as the mechanism for avoiding lysosomal degradation^28^. It seems phagosome maturation goes hand-in-hand with autophagosome maturation and that at least *Mtb*-containing autophagosomes do not mature into autolysosomes without recruiting RAB7 to form an intermediate amphisome. How the resident virulent *Mtb* could block autophagosome fusion with late endosomes remains unanswered. However in case of phagosome maturation, it was shown that the failure to recruit RAB7 to H37Rv phagosome was contingent on the lipid product phosphatidylinositol 3-phosphate (PtdIns[3]P) and subsequent binding of RAB5 effector EEA1^29^. However it remains unclear if that holds true even in the case of autophagosomes. Interestingly a number of studies propose, in other systems, role of RAB7 in the maturation phase of autophagy^30^,^31^,^32^. The importance of maturation step of autophagy in case of *Mtb* infections was also supported by the study where TBK1, an innate immunity regulator was shown to regulate autophagy maturation to eliminate BCG infections^15^. Intriguingly, upon RAB7 depletion in THP-1 macrophages, even the general autophagy flux gets perturbed. That raises another question on how the selectivity in autophagosome maturation could be ensured by resident virulent *Mtb* if RAB7 is essential for autophagosome maturation in general. One plausible explanation for this may be selective modulation of RAB5 effector function by H37Rv leading to altered autophagosome. Thus even though late endosomes could efficiently fuse with other autophagosomes, H37Rv-containing autophagosomes evade this process. Such mechanisms are studied in case of phagosome maturation^29^, whether similar mechanism could be functional in case of autophagy needs further investigations. In this study we could not distinguish autophagy from LC3 associated phagocytosis (LAP). Involvement of LAP in *Mtb* infections remains debatable despite some reports emerging on it, and needs further investigations^33^,^34^. The crosstalk between the autophagy pathway and the phagosome maturation pathway has been schematically summarized in Fig. 5.

In conclusion we report here a mechanism whereby virulent *Mtb* strain could selectively block maturation of the resident autophagosomes. We show that the virulent *Mtb* could achieve this by avoiding RAB7 recruitment to form the amphisomes. The crucial role of amphisome compartment in the regulation of intracellular *Mtb* survival has added new dimensions to our understanding of cell autonomous defense mechanisms in case of *Mtb* infections. Understanding the regulation of autophagy maturation at molecular level could help identify novel and perhaps better host directed drug targets against tuberculosis.

## Materials and Methods

### Ethics statement

All the experiments with animals were performed in accordance with the approved guidelines and regulations of the Institutional Animal Ethics Committee (IAEC). The experiments were performed upon prior approval from the institutional ethics committee (IAEC) of International Center for Genetic Engineering and Biotechnology (Approval no.: ICGEB/AH/2013/03/IMM-38).

### Cells and Infections

THP-1 cells were differentiated with PMA (Sigma, P1269) at a concentration of 20 ng/ml for 24 hours. For infection, *Mtb* (H37Ra and H37Rv) single-cell suspensions were prepared as described by Kumar *et al.* and infections were done at an MOI of 1:10 for 4 hours followed by amikacin treatment of 2 hours. The subsequent time points were considered from here on.

### Bacterial cultures and media

Bacteria were grown as stationary cultures in Middlebrook 7H9 broth (BD Difco, Becton Dickinson) supplemented with 10% ADC (Becton Dickinson), 0.4% Glycerol and 0.05% Tween-80 until the mid-log phase. The bacteria were then harvested, washed with RPMI and used for suspension preparation.

### Bacterial suspension preparation and Infection

Bacterial whole cell suspension was dispersed by aspiration five times each with a 23- and then a 26-gauge needle, followed by an additional dispersion by aspiration 3 times through a 30-gauge needle. The dispersed bacteria were allowed to stand for 5 min to allow the clumps to settle down. The upper half of the suspension was then used for the experiments. Quantitation of bacteria was done by taking absorbance at 600 nm wavelength (0.6 O.D. corresponds to ~100 × 10^6^ bacteria). Complete media containing the required number of bacteria was added to the cultured cells at an MOI of 10, followed by a short spin of the culture plates at 700 rpm for 5 minutes. After 4 hours of infection, the cells were washed twice with warm RPMI and treated with Amikacin (200 μg/ml, HiMedia laboratories) to remove any extracellular bacteria. After two hours of treatment, the cells were washed and maintained in complete media. For microscopy experiments, the desired number of bacteria were stained with PKH67 (green)(Sigma-Aldrich Co. MINI67-1KT), lipophilic fluorescent dye, as per the manufacturer’s protocol. The stained bacteria were passed thrice through a 26-gauge needle and were then used for infection.

### Animals and isolation of BMDMs

Bone marrow derived macrophages (BMDMs) were isolated from femurs of BALB/C mice (4-6 weeks old, female). BMDMs were obtained by culturing the marrow cells in the presence of macrophage-colony stimulating factor (M-CSF, eBioscience, 14-8983-80) for 7 days. Fully differentiated macrophages were harvested and seeded for infection with H37Ra or H37Rv. The infection protocol was same as described above for THP-1 macrophages.

### Inhibitor treatments

E64-d (Sigma, E8640) and PepstatinA (Sigma, P5318) treatments were given at a concentration of 25 μg/ml and 10 μg/ml, respectively for 2 hours prior to fixation. Autophagy was inhibited by 3-Methyladenine (Sigma, M9281) treatment, added 48 hours post infection, at a concentration of 5 mM. Autophagy flux was monitored using BafilomycinA1 (Invivogen, tlrl-baf1) at 100 nM for 3 hours. LysoTracker Red DND-99 (Molecular Probes, L-7528) was used to monitor acidified lysosomes at 200 nM concentration for one hour.

### Construction of PhoP over-expressing *Mtb* H37Ra strain

For expressing H37Rv PhoP in H37Ra, gene encoding H37Rv PhoP (Rv 0757) was PCR amplified and cloned into E.coli-Mycobacterial shuttle vector, pSD5hsp^35^. The pSD5-*phoP* construct was then electroporated into *Mtb* H37Ra and transformants were selected on 7H11 medium containing kanamycin (25 ug/ml). The expression of phoP was under strong *hsp65* gene promoter from *M. leprae*.

### siRNA treatments

We followed established protocols for siRNA treatments of differentiated THP-1 cells as described earlier^10^. *RAB7* siRNA were procured from Dharmacon (M-010388-00-0005). Addition of RAB7 siRNA was done after amikacin wash and treatment was done for 48 hours.

### Confocal Microscopy

One hour prior to the termination of the experiment, complete media containing LysoTracker dye at a concentration of 200 nM was added to the cells for staining of acidified lysosomes. Following the required treatments, the cells were fixed with 4% paraformaldehyde (Sigma) for 20 minutes, followed by 3 washes with 1X PBS. The cells were permeabilized using 0.2% (w/v) TritonX-100 in 1X PBS for 20 minutes. Blocking was performed using 3%(w/v) BSA and 0.5%Tween20 in 1X PBS for one hour. This was followed by primary and secondary antibody staining for duration of one hour each. The primary antibodies used in the study are LC3 (Novus, NB100-2220, Cell Signaling Technology, 2775S), RAB7 (Santa Cruz Biotechnology, SC-6563). Commercially available Alexa dye conjugated secondary antibodies (Molecular Probes, A31556 and A11057) were used for immunostaining. Antibody dilutions were made in blocking solution as per the supplier’s protocol. The coverslips were washed thoroughly with 1X PBS and were mounted onto glass slides with Antifade reagent (Invitrogen Molecular Probes). Images were acquired randomly from each set of stained cells with a Nikon EclipseTi-E laser scanning confocal microscope equipped with a 60X/1.4NA PlanApochromat DIC objective. Images were acquired using the softwares EZ-C1 3.80 and NIS-Elements. Image analyses were performed using the software Imaris version 7.6.4 (BitPlane).

### Image Capture and analysis

Stained cells were observed with a Nikon Ti-E microscope equipped with 60X /1.4 NA planapochromat DIC objective lens. Samples were excited at 543 nm with He-Neon laser, 488 nm with an Argon ion laser and in the UV range with a blue diode. Images were acquired sequentially to avoid bleed-through signal, with a scanning mode format of 512 × 512 pixels. The transmission and detector gains were set to achieve best signal to noise ratios and the laser powers were tuned to limit bleaching of fluorescence. The refractive index of the immersion oil used was 1.515 (Nikon Corporation). All settings were rigorously maintained for all experiments. All images were qualitatively assessed using Imaris version 7.6.4 (BitPlane). All the images are in the Tiff RGB 24 format. M1 co-localization coefficient of *Mtb* and various markers were calculated in the software. The percentage of *Mtb* in autolysosomes and amphisomes were estimated by calculating the percentage of *Mtb* voxels in LC3:Lysotracker and LC3:RAB7 colocalized channels. The results represent an average of at least three independent sets of experiments.

### Statistical analyses

Statistical analyses were performed using Student’s t-tests (two-tailed) with ‘*’ and ‘**’ representing significant difference between the tested group at 95% (p < 0.05) and 99% (p < 0.01) level of confidence.

## Additional Information

**How to cite this article**: Chandra, P. *et al.* *Mycobacterium tuberculosis* Inhibits RAB7 Recruitment to Selectively Modulate Autophagy Flux in Macrophages. *Sci. Rep.* **5**, 16320; doi: 10.1038/srep16320 (2015).

## Supplementary Material

Supplementary Information
