# Supplementary material for: Mycobacterium tuberculosis Inhibits RAB7 Recruitment to Selectively Modulate Autophagy Flux in Macrophages
Source: Sci Rep. 2015 Nov 6;5:16320. doi: 10.1038/srep16320 (PMC4635374; doi:10.1038/srep16320)

Supplemental Information for the Manuscript "***Mycobacterium tuberculosis* Inhibits RAB7 Recruitment to Selectively Modulate Autophagy Flux in Macrophages**"

Pallavi Chandra[1], Swapnil Ghanwat[1], Sumit Kumar Matta[1], Swati Seth Yadav[1], Mansi Mehta[1, 2], Zaved Siddiqui[1], Amit Singh[2] and Dhiraj Kumar[1]*

[1] Immunology Group, International Centre for Genetic Engineering and Biotechnology, Aruna Asaf Ali Marg, New Delhi-110067, India

[2] Department of Microbiology and Cell Biology, Centre for Infectious Disease Research, Indian Institute of Sciences, Bangalore-560012, India

Serial No.	Supplemental Item	Title
1	Supplemental Figure S1	Survival of H37Ra and H37Rv in THP-1 macrophages
2	Supplemental Figure S2	Characterisation of the H37Ra:PhoP strain
6	Supplemental Figure S3	Role of RAB7 in autophagosome maturation.

Figure Legends

Supplemental Figure S1: Survival of H37Ra and H37Rv in THP-1 macrophages

PMA differentiated THP-1 macrophages were infected with H37Ra and H37Rv. At different time points post-infection, cells were lysed and plated onto OADC agar plates for Mtb colony forming units (CFU) count. Panel A shows CFU of H37Ra (blue) and H37Rv (red) at various time points post-infection in THP-1 macrophages (values are mean ± SD). For panel B, flow cytometry was performed to analyse direct antibody binding to the *Mtb* after staining with the following antibodies unstained Mtb (left), LC3 rabbit polyclonal antibody followed by Alexa 488-conjugated IgG (H+L) goat anti-rabbit secondary antibody (middle) and Mtb-specific rabbit polyclonal antibody (ab905 from Abcam) followed by Alexa 488-conjugated IgG (H+L) goat anti-rabbit secondary antibody (right). For panel C, THP-1 cells were infected with Mtb and confocal microscopy was performed to analyse non-specific antibody binding to the bacteria. Shown are images from (left to right) Mtb labelled with PKH green dye, Alexa 405-conjugated IgG (H+L) goat anti-rabbit secondary antibody, merged image and DIC (Scale bar=10μm).

Supplemental Figure S2: Characterisation of the H37Ra:PhoP strain

Panel A shows restoration of ESX1 system in the PhoP complemented H37Ra strain. Total RNA isolated from H37Ra and PhoP complemented H37Ra were subjected to real-time PCR using gene specific primers mentioned in the figure to check their relative expression in the two strains. The genes selected are known to be dependent on mycobacterial ESX1 system, which is compromised in the H37Ra strain due to mutations in the PhoP gene.

Supplemental Figure S3: Role of RAB7 in autophagosome maturation

Western blots showing specific knock-down of RAB7 by siRNA treatment in THP-1 macrophages (S3A). Samples were harvested 24 hours post siRNA treatment for Western blot. LC3 Western blot showing effect of RAB7 knockdown on general autophagy flux in THP-1 macrophages 24 hours post siRNA addition is shown in S3B. Corresponding plots showing autophagy flux in RAB7 siRNA treated versus untreated THP-1 macrophages are shown in S3C (Value± SD).

Supplemental Figure S1

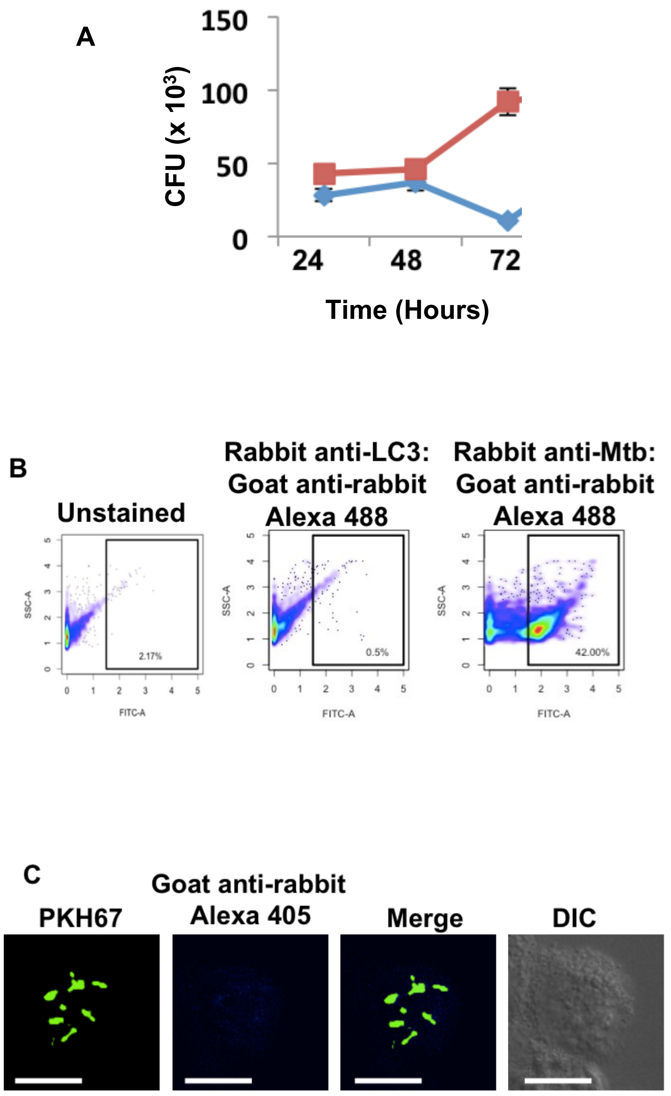

Supplemental Figure S2

A

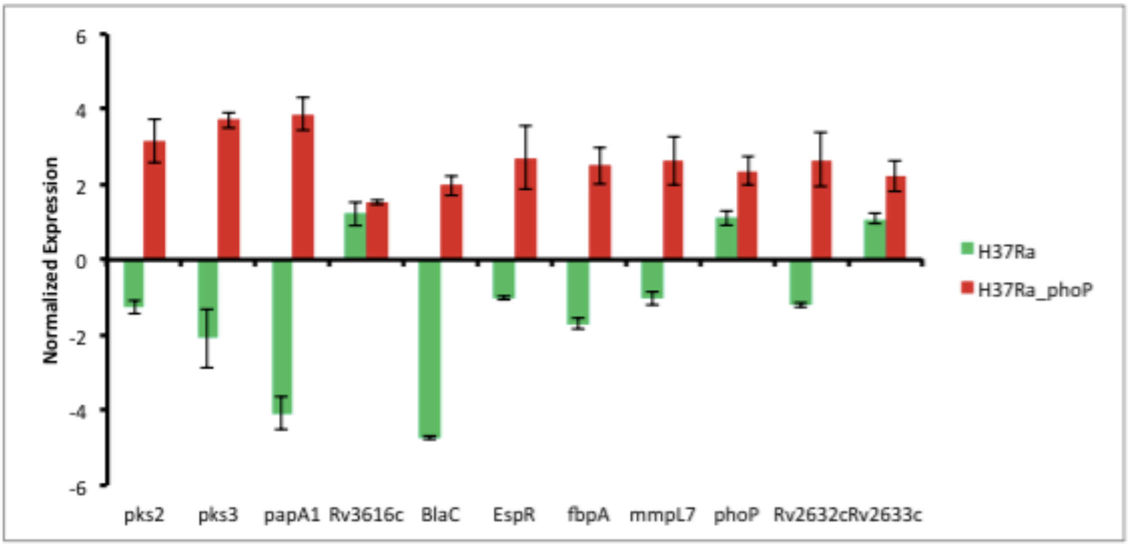

Supplemental Figure S3

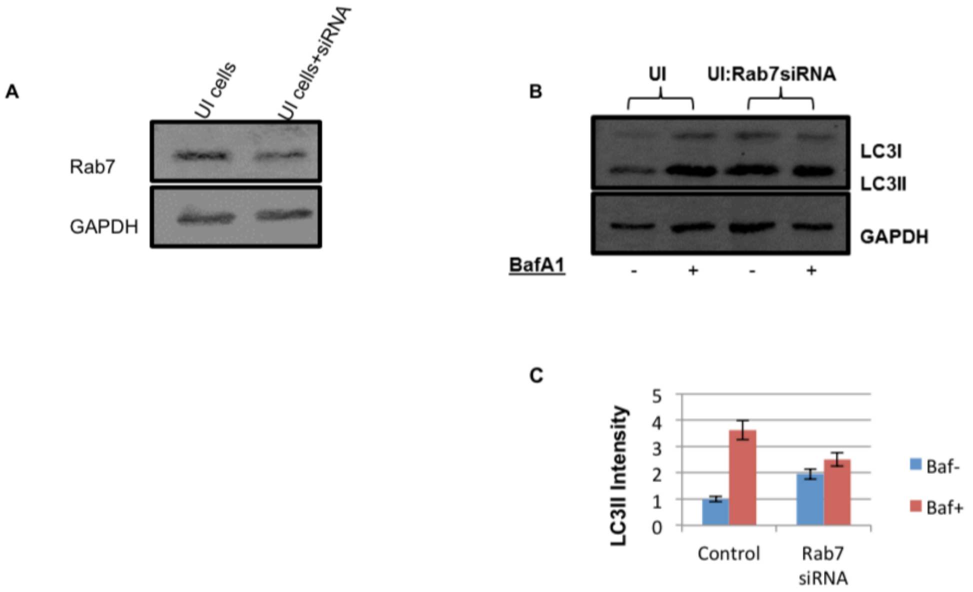

Supplement: Supplementary Information [file srep16320-s1.pdf]
